# Parametric Construction of Episode Networks from Pseudoperiodic Time Series Based on Mutual Information

**DOI:** 10.1371/journal.pone.0027733

**Published:** 2011-12-22

**Authors:** Frank Emmert-Streib

**Affiliations:** Computational Biology and Machine Learning Lab, Center for Cancer Research and Cell Biology, School of Medicine, Dentistry and Biomedical Sciences, Queen's University Belfast, Belfast, United Kingdom; University of East Piedmont, Italy

## Abstract

Recently, the construction of networks from time series data has gained widespread interest. In this paper, we develop this area further by introducing a network construction procedure for pseudoperiodic time series. We call such networks *episode networks*, in which an episode corresponds to a temporal interval of a time series, and which defines a node in the network. Our model includes a number of features which distinguish it from current methods. First, the proposed construction procedure is a parametric model which allows it to adapt to the characteristics of the data; the length of an episode being the parameter. As a direct consequence, networks of minimal size containing the maximal information about the time series can be obtained. In this paper, we provide an algorithm to determine the optimal value of this parameter. Second, we employ estimates of mutual information values to define the connectivity structure among the nodes in the network to exploit efficiently the nonlinearities in the time series. Finally, we apply our method to data from electroencephalogram (EEG) experiments and demonstrate that the constructed episode networks capture discriminative information from the underlying time series that may be useful for diagnostic purposes.

## Introduction

The definition and the study of discrete objects in the form of graphs or networks with specific properties is a topic that reaches back over two hundred years [1]–[3], eventually leading to the founding of graph theory [4]–[6]. Despite the mathematical origin of this field, its contemporary form, sometimes called network analysis [7]–[10], attracts interdisciplinary interest as networks can be found pervasively in nature. For this reason many new methods have been developed in recent years to provide quantitative approaches for the structural analysis of complex networks [11].

One specific aspect of network analysis that is currently of great interest is the inference, reconstruction and construction of networks from data. For example, in molecular biology and the biomedical sciences powerful experimental assays allow the measurement of the activity of genes or gene products on a genome-scale. Several methods have been introduced to infer various forms of gene networks [12] from such high-throughput data [13]–[20]. Similarly, in neuroscience one tries to infer neural networks that capture the interactions among neurons or neuronal regions [21]–[23]. The ultimate goal of these methods is to infer causal networks [24], [25]. That means in the above networks an interaction in the inferred networks corresponds to a predicted physical interaction among system variables that can be verified experimentally [26]. For example, in gene networks this could correspond to the binding of two proteins or for neural networks this could be the synaptic connection of two neurons. In other words, it is assumed that there exists a network that underlies the data which shall be estimated or reconstructed from the data. Recently, a fundamentally different way to construct networks, using time series data, has been introduced [27]–[34]. The principle difference to the methods discussed above is that the networks constructed this way are merely a *representation* of the data. That means it is not assumed that there exists a network that is behind the data which should be reconstructed, but the network is *constructed* from the data to form a formal representation thereof, which serves as a means for further analysis. In the remainder of this paper we are concerned with the latter type of networks.

Specifically, there are two network construction methods for time series data that have gained considerable popularity since their introduction. The first network construction method generates so-called *cycle networks* [34]. For this method a node in the constructed network corresponds to a cycle in the time series and two nodes are connected if the corresponding cycles are similar to each other as measured by a correlation coefficient. That means the correlation coefficients between pairs of cycles give the components of a similarity matrix which is used to obtain the connectivity of the cycle network. The resulting network is undirected, because the correlation coefficient does not provide information about a directionality. It is noted in [34] that the similarity matrix can be either filtered by applying a global threshold parameter transforming it into a binary network or the similarity matrix can be used unfiltered in which case it is a fully connected network. The key of the above method is that each cycle corresponds to a well defined part of the time series, which can be seen as a profile vector of a certain length. The second of these methods constructs so-called *visibility graphs* from a time series [30]. In a visibility graph, nodes correspond to time points of a time series and two nodes are connected if there is a certain criterion met that involves the values of the time series. A visualization of this criterion shows that two nodes are connected if one time point is *visible* from the second one, hence the name of these networks. This leads to an undirected and unweighted network.

The major purpose of this paper is to introduce a construction procedure for networks from pseudoperiodic time series. Here, by pseudoperiodic we mean a time series that exhibits oscillatory or even chaotic behavior. Our construction method adds on previous methods, and includes several key features that makes it distinct. The first feature of our method is to *estimate* the connectivity structure of the constructed network from the underlying time series. This is different to the construction of visibility graphs [30] that establish the connectivity among nodes by testing a *geometric* criterion, instead of a statistical one. However, this is similar to cycle networks [34] that estimate the correlation coefficients between cycles. Also financial networks have been constructed based on the estimation of correlation coefficients [29]. Considering the fact that we assume a time series to behave oscillatory or even chaotic one can expect that this time series is strongly nonlinear. For this reason we estimate mutual information values, instead of correlation coefficients, because the mutual information is capable of capturing nonlinear effects in a time series [35], [36] and, hence, provides more accurate estimates of the similarity of nonlinear time series intervals. Also, this builds directly on results obtained from other fields in which estimates of mutual information values have been used to infer causal gene networks [14], [37], [38]. Second, we define a node in the constructed network as an *episode*. An episode is a temporal interval of the time series that consists of 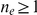 consecutive cycles. That means an episode is 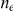 times longer than a cycle. The extended length of an episode, compared to a cycle, has the advantage of increasing the accuracy of the statistical estimates of the mutual information value. The reason for this is that a cycle does not need to have a certain minimal length to qualify as a cycle. However, it is clear that very short cycles convey less information about the time series than long cycles. Due to the fact that the notion of a ‘cycle’ is parameter free, one cannot adjust for this shortcoming. For this reason we extend the principle idea behind the usage of a cycle in the construction of a network [34] by means of an episode. Third, our network construction model is a parametric method because an episode is a function of 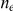, the number of consecutive cycles. This gives us a parameter that can be optimized to result in the ‘best’ network for a given time series. We call the optimal value of 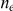 the *effective length* of an episode and provide a procedure to estimate its value. None of the previous methods introduced to construct networks from time series data is parametric. Fourth, the size of the constructed network, which corresponds to the number of nodes, is adjustable in our model. Again, this is related to the length of an episode. For the *effective length* of an episode, this results in networks of minimal size, which means that it consists of the least number of nodes.

This paper is organized as follows. In the next section we introduce episode networks and their construction. Then we present results studying the influence and the distribution of the mutual information values of episode networks. Next, we compare properties of episode and cycle networks. We then show several examples to demonstrate how the effective length of an episode can be estimated. Finally, we apply our method to EEG data. By this analysis we demonstrate that the constructed episode networks capture discriminative information from the underlying time series that might be useful for diagnostic purposes. We finish this article with a summary and conclusions.

## Methods

In this section we introduce and discuss the construction procedure for episode networks. Further, we introduce an algorithm to estimate the *efficient length* of an episode to construct such networks.

### Construction of episode networks

Episode networks, defined below, are based on mutual information values [39], [40]. The mutual information is a measure for the nonlinear dependency of two random variables 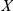 and 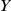, defined by
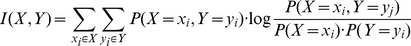
(1)Here by 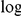 we mean the logarithm to the base 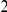. 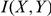 is always 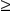
0. If the two random variables are independent from each other the mutual information becomes zero, because 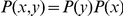.

**Figure 1:**
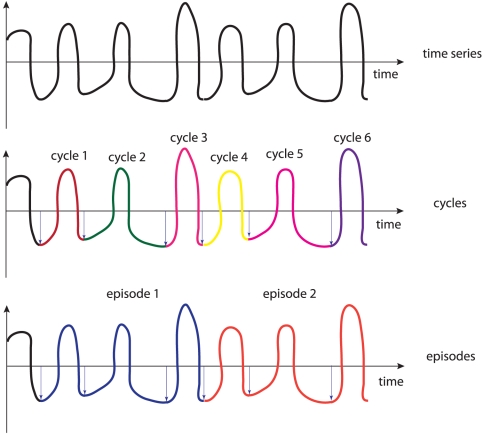
Top: Hypothetical time series. Middle: Identification of all cycles in the time series. Bottom: Definition of episodes. In this case, an episode consists of three consecutive cycles.

Before we define the construction procedure for episode networks formally, we provide a brief depiction of it. The basic idea that underlies the networks we want to construct is as follows. For a given pseudoperiodic time series that consists of 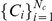 cycles we define an episode as 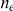 consecutive cycles. This results in 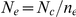 different episodes 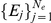. That means an episode is an interval of a time series that contains 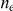 consecutive cycles. This is visualized in Fig. 1. We use these episodes as the nodes in a network. The connection of this network is based on the similarity between these episodes. Here we measure the similarity between pairs of episodes by their mutual information value [39], [41], 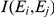. That means we estimate a similarity matrix 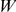, whose components correspond to the mutual information values between pairs of episodes, i.e., 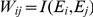. From the similarity matrix 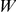 we construct an episode network as the *maximal connected component*, which is an undirected, unweighted network. Here we define the *maximal connected component* as the network 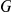 obtained from 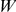 which is (1) a connected network and (2) the edges used to construct 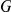 have maximum mutual information values. The first property means that in an episode network each node is connected via an undirected path to any other node. The second property defines its construction procedure from 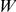 which is as follows: First, we initialize the adjacency matrix 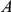 of 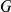 as a zero matrix. Then we identify the largest edge weight (mutual information value) in 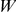 and its corresponding episode pair 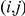 and add an undirected, unweighted edge in 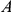, i.e., 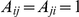. If this results in a connected component of the vertices in 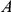 we stop, otherwise we proceed to the next largest edge weight in 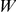 and continue until we obtain a connected component of the vertices in 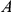. Formally, our construction corresponds to a *greedy optimization* [42] of the mutual information values used to construct 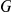.

**Figure 2:**
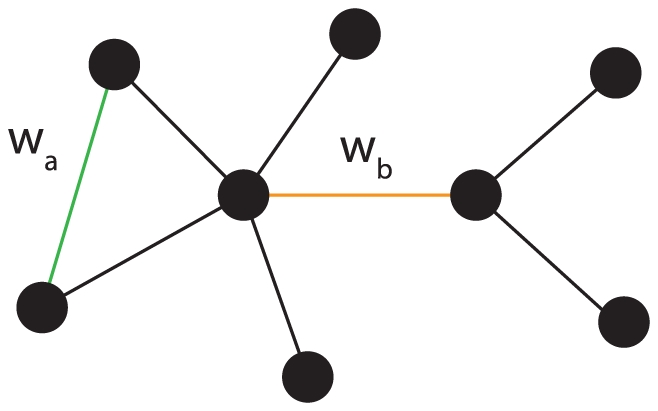
Construction principle based on the maximal connected component. If 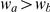, edge 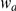 will be added, otherwise 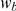. This is in contrast to the MST algorithm which would always add 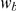.

The construction of the maximal connected component is visualized in Fig. 2. Assuming the black edges have already been added and all other edge weights are zeros, except 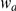 and 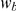, for which holds 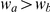. In the next step we face a decision which of the two edges 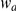 and 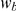 to include. According to our construction procedure, the edge 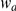 will be added because its mutual information value is larger than 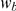. This is in contrast to the *minimum spanning tree* (MST) algorithm [42] which would add the edge 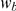 instead of 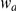. That implies that the resulting network we obtain from our procedure does not need to be a tree, but the network can have an arbitrary complex structure.

The construction procedure of an episode network from a time series can be summarized by the following four steps.

**Algorithm 1** construction of an episode network

1: Given: pseudoperiodic time series
2: Initialize: adjacency matrix 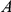 as zero matrix
3:. Initialize: episode length 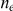 as a natural number
4: identify all cycles in the time series, 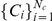

5: construct 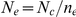 episodes from the cycles, 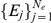

6: **for** 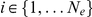 **do**
7: **for** 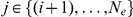 **do**
8: 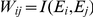 - estimate mutual information
9: **end for**
10: **end for**
11: **while** 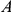 is not connected **do**
12: 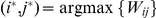

13: 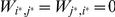

14: 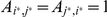

15: **end while**
16: Return: adjacency matrix 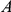 of the episode network
  1. Identify the cycles 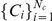 in the time series.
  2. Define the episodes 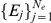 in the time series. An episode corresponds to a node in the constructed network 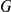.
  3. Estimate the similarity between pairs of episodes 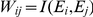 by their mutual information value.
  4. Connect nodes (episodes) in the network 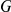 if they are part of the maximal connected component in 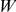.

Formally, the construction procedure for episode networks is defined in algorithm 1. We want to point out that, usually, the length of different cycles 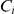 will be different, hence, the length of different episodes 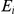 will be different too. In order to estimate the mutual information value between episode pairs of different length, we employ a similar strategy as in [34], [43] used for correlation coefficients. That means if episode 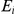 is longer than 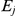 we estimate all possible mutual information values one can obtain by shifting the start position 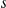 of 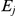 with respect to 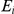 and select from these values the maximum mutual information value, i.e.,
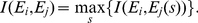
(2)This allows to circumvent the problem of unequal episode lengths.

Our network model to construct episode networks is similar to the construction of cycle networks [34] but has the following benefitial features. First, due to the fact that the model is intended to convert a pseudoperiodic time series into a network, one can assume that the signal in the time series is strongly nonlinear. For this reason it appears sensible to estimate the similarity between episodes with a measure that is capable of capturing such nonlinearities. The mutual information is a nonlinear extension of the correlation coefficient between two random variables and, hence, possesses this property [35], [36]. Further, it has been demonstrated for large-scale high-throughput data from gene expression experiments that mutual information based inference methods are able to reconstruct molecular interactions among genes or gene products reliably [14], [37], [38], [44], [45]. This demonstrates that theoretical properties of the mutual information translate to data from experiments making this measure a favorable choice over correlation coefficients.

Second, a cycle is a well defined entity within a pseudoperiodic time series [46] and as such is appropriate to represent a node of a network. However, this bears an implicit limitation with respect to the length of the profile vectors that are compared. The problem is that from simulation studies we found that the estimated correlation coefficients between two cycles is in general very high, and frequently even close to 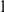. One reason for this is that for pseudoperiodic time series the length distribution of the profile vectors is of similar order. Another reason is the ‘periodic shape’ of the cycles, which they naturally represent. Both effects do not prevent, but hamper that the full range of possible correlation coefficients from 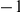 to 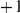 is actually observed. The usage of an episode as basic building block of a node, and of the profile vectors, leads naturally to larger profile vectors and enables by this a larger diversity of observed similarity values among different episodes.

Third, an episode network grows proportional to the number of episodes 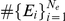 in the time series. That means, the number of nodes in an episode network grows with the number of episodes, 
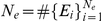
, and not with the number of cycles 
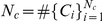
 as the cycle networks. Due to the fact that an episode consists of 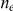 cycles we obtain 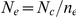. That means the size of an episode network is directly controllable by the number of cycles 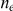 that define an episode. Further practical implications of this relate back to point 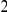 discussed above as well as to a more efficient computational complexity of an analysis of smaller compared to larger networks.

The last point raises the question how to choose the length of an episode to construct episode networks, which are best suited for a given time series. This point is addressed in the next section.

### Effective length of an episode

First, we would like to note that in order to define a procedure to determine the effective length of an episode we need to specify a measure 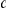. This measure will serve as a reference that allows us to quantify what we mean by *effective*. Because the length of an episode has an influence on the structural properties of the resulting episode network 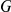, we are looking for a measure to quantify the structural properties of a network, i.e., 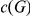. Potentially, there are several choices for such a measure. For example, the mean path length or the edge density of a network are possible measures. Due to the fact that we will study the value of this measure for different values of the episode length, 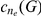, we require it to be largely independent of the size of the network, because 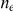 effects directly the size of the constructed episode network. For our analysis we use the global clustering coefficient as measure. The global clustering coefficient, also called global transitivity, is a well-known measure that captures an important property of complex networks [47]. Briefly, it measures the probability that adjacent nodes of a vertex are connected with each other. For our analysis we use the global clustering coefficient of a network, 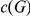, which is defined as the average clustering coefficient of all individual nodes in the network. In the following definition we specify the notion of the *effective length* of an episode. In this definition, the function 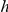 is directly related to the measure 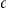 as will be explained in more detail below.

**Definition 1** (*effective length*) *As effective length of an episode we define the maximal length of an episode, denoted as* 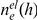, *for which the structural properties of a population of episode networks is maximal, as measured by* 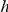. *Quantitatively, we define* 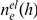 *as*

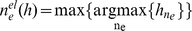
(3)
*with respect to the function* 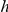.

In order to point out that the effective length 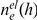 of an episode network is defined with respect to a network measure, 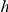, we included this dependency in the above definition explicitly for reasons of clarity. The efficient length is the maximum of 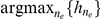 because in case there are several elements that maximize 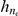, we want to chose the largest episode length because this results in the smallest networks.

**Algorithm 2** procedure to estimate the effective length 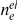 of an episode

1: Initialize:
2: 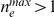

3: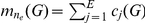 for 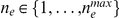

4: 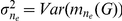 for 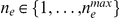

5: 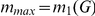 - mean global clustering coefficient for 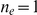

6: 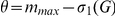

7: 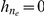 for 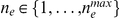

8: 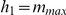

9: **for** 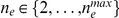 **do**
10: **if** 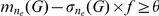 **then**
11: 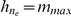

12: **else**
13: 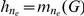

14: 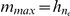

15: 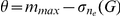

16: **end if**
17: **end for**
18: Return:
19: 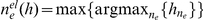

The rational behind the above definition of the optimization criterion is to select the length of an episode in a way that provides us with the *maximal* information from an episode network of *minimal* size. The information is maximal because for 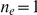 an episode network consists of the maximal number of nodes, 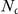, and, hence, can exhibit its largest structural diversity. This can be seen as the highest resolution achievable. Increasing the value of 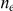, leads to a reduction of the number of nodes in an episode network and, potentially, to a restriction in the diversity of the network structure. However, by searching the maximum of 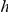 and 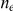, we obtain smaller episode networks that represent approximately the same structural information as larger networks. Hence, using the effective length 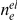 leads to the smallest size of an episode network with similar structural properties as larger episode networks, constructed from the same time series.

The discrete function 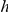 in the above definition is obtained from estimates of structural properties of 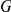 in dependence on 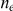. Formally, we define 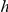 in algorithm 2. In this procedure, 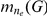 corresponds to estimates of the mean global clustering coefficients of a population of episode networks and 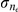 to the standard deviations. Practically, we approximate this population of networks by an ensemble of networks of size 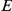. The basic idea underlying the definition of the function 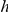 is to utilize information about the variability of the structural properties of networks from the same population. It is necessary to formulate this with respect to a population, because every structural property of an episode network, e.g., the clustering coefficient 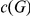 is a random variable, due to the fact that the time series used to construct 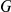 is just a sample from a dynamical system. In order to assess such a random variable one needs to consider its inherent variability. As long as 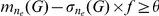 holds, the values of the constructed episode networks are within one standard deviation of the threshold 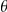. Here the factor 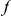 allows to adjust this range, but for our simulations we used 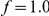. According to the central limit theorem, for a sufficiently large episode network the values of the global clustering coefficients 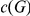 are approximately normal distributed (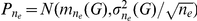) with mean 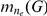 and variance 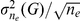 [48], because the global clustering coefficient is the average of the clustering coefficients of the individual nodes in the network. Considered from this perspective, the criterion 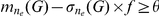 means that the probability to observe a value of 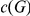 larger or equal to 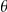 is 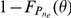. Here 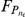 is the cumulative distribution function of 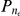. Hence, our procedure identifies the maximal length of an episode 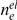 for which the structural properties of episode networks are still within the variability range of the population of episode networks.

## Results

In the following we, first, study the influence and the distribution of the mutual information values of episode networks and compare properties of episode and cycle networks [34]. Then we show how to determine the effective length of an episode to construct the networks. Finally, we apply our method to EEG data to demonstrate that the constructed episode networks capture discriminative information from the underlying time series.

### Influence and distribution of the mutual information

**Figure 3:**
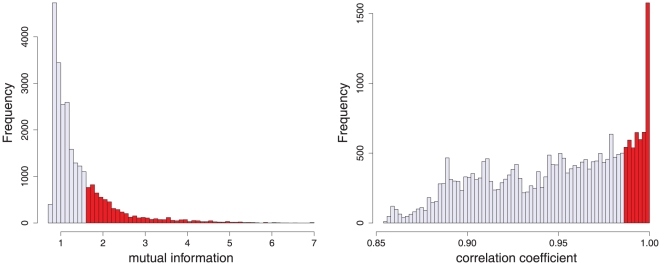
Histogram of mutual information values (left) and correlation coefficients (right) obtained for a time series with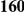 episodes and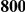 cycles generated with a Rössler system. Only the values colored in red are used to construct the corresponding episode and cycle network.

For the following analysis we use time series data generated with a Rössler system [49] given by
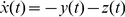
(4)

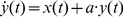
(5)

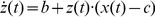
(6)For parameter values of 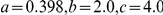 the Rössler system exhibits a chaotic behavior. From this system, we use the x-component to generate a time series with 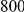 cycles. For 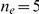 we obtain 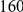 episodes. From this we estimate an episode and a cycle network 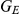 and 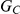. First, we want to note that the episode network contains 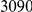 edges and the cycle network contains 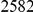 edges. Already from these numbers one gets the impression that the usage of the mutual information as estimator of episode similarity has a profound impact on the inferred network structure. In order to demonstrate this more clearly, we show in Fig. 3 the histograms of the mutual information values (left figure) and the correlation coefficients (right figure) of all none-zero edge weights of the mutual information matrix and the correlation matrix. From this follow two interesting observations. First, the distribution of mutual information (MI) values appears vertically mirrored compared to the distribution of the correlation coefficients. That means, if one goes from high to small values of the MI values, one enters first the long tail of the distribution and then reaches the center of mass of the majority of values. For the correlation coefficients this behavior is reversed. Due to the fact that our network construction procedure adds successively edges starting with high edge weights (mutual information values) and working toward lower values, the distributional shape of the mutual information values is beneficial because it allows for a more selective procedure.

The second related observation refers to the covered range of selected values, colored in red in Fig. 3. In this figure, the mutual information (MI) values and the correlation coefficients that were actually used to construct 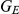 and 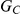 are colored in red. For the episode network the range covered by the selected values is 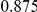 = (maximal value of the selected MI values - minimal value of the selected MI values)/(maximal value of all MI values - minimal value of all MI values) whereas for the cycle network the covered range is only 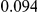. This is intimately connected with the distributional shape of both networks, as discussed above, and the location of its tail. To quantify the distributional shape of the tail of the mutual information values we conduct a statistical test suggested in [55] to test if the tail follows a power law 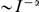. A maximum-likelihood fit results in an exponent of 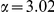 and a goodness-of-fit test (Kolmogorov-Smirnov) gives a p-value of 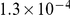, indicating that the tail of the mutual information values is unlikely to follow a strict power law. That means the distribution has a long tail but does not exactly decay as 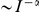.

The next dynamical system we study is a Duffing map [50],
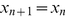
(7)

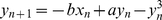
(8)For the parameters 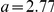 and 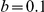 the obtained time series is chaotic. Using this parameter configuration we generate a time series with 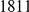 episodes (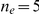). A maximum-likelihood fit of the tail of the mutual information values gives an exponent of 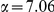 and the goodness-of-fit test gives a p-value of 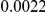 [55]. This test shows that the tail of mutual information values for the Duffing map is closer to a power law than the Rössler system, but is also unlikely to be an exact power law, 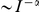.

**Figure 4:**
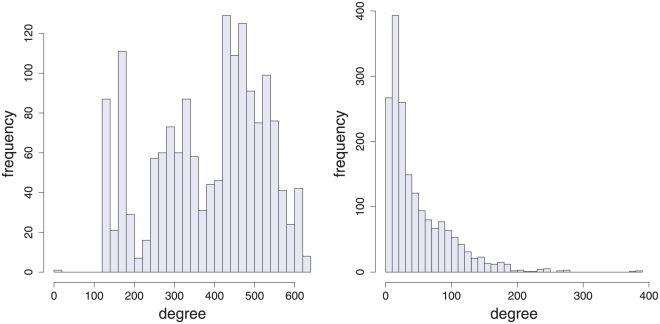
The degree distribution of episode networks. Left: Rössler system with 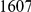 episodes. Right: Duffing map with 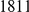 episodes.

To demonstrate that the resulting episode networks for a Rössler system and a Duffing map have a different network connectivity, we show in Fig. 4 the degree distribution of the episode networks (left: Rössler system 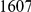 episodes; right: Duffing map with 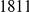 episodes). Despite the fact that we used parameters of the Rössler system and the Duffing map leading to a chaotic behavior of both time series, the resulting degree distributions of the episode networks are quite different from each other. This means that not every chaotic system maps to a network with the same connectivity structure. Despite the fact that the mutual information distribution of an episode network of a Rössler system has a long tail, its degree distribution has not. This is similar to the results obtain for cycle networks [34].

**Figure 5:**
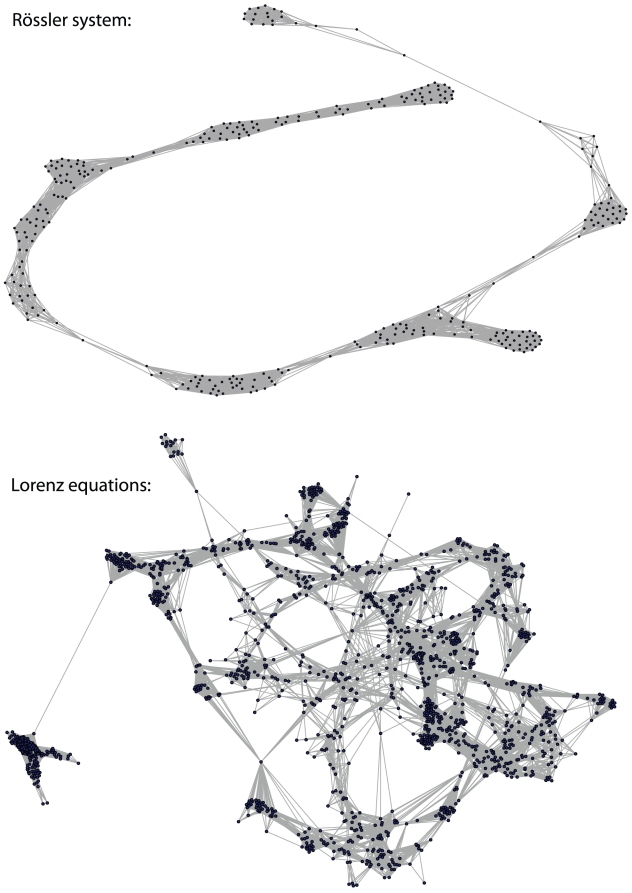
Examples of episode networks. Top: The network was constructed from a Rössler system and consists of 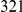 nodes. Bottom: The network was constructed from Lorenz equations and consists of 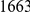 nodes.

In Fig. 5 we show two visualizations of episode networks. The top network is obtained from a Rössler system consisting of 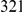 nodes and 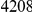 edges. Its average path length is 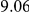, the clustering coefficient is 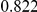 and the maximal degree is 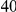. The second network is obtained from a Lorenz system,
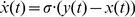
(9)

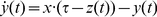
(10)

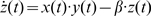
(11)with the parameters 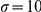, 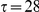 and 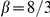, which lead to a chaotic time series. The episode network was constructed using the x-component of the Lorenz equations and consists of 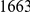 nodes and 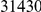 edges. Its average path length is 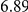, the clustering coefficient is 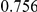 and the maximal degree is 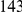. Both networks are obtained for 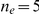. It is interesting to see that despite the fact that both time series are chaotic the resulting episode networks ‘look’ quite different, which reflects also in their structural properties. We want to emphasize that the shown episode networks are obtained by the application of algorithm 1. That means, there is no manual adjustment of any parameter necessary.

**Figure 6:**
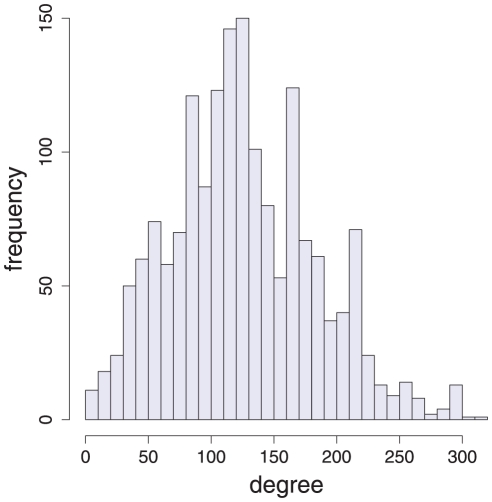
The degree distribution of the episode network constructed from the Lorenz equations, shown in **Fig. 5**.

In Fig. 6 we show the degree distribution for the episode network constructed from the Lorenz equations, shown in Fig. 5. Comparing this degree distribution with the ones for the Rössler system and the Duffing map, shown in Fig. 4, one can see that also the episode network constructed from the Lorenz equations has a characteristic connectivity structure that is different to the other two dynamical systems. This confirms also the visual impression from the graphical representation of the episode networks shown in Fig. 5.

### Estimation of the effective length of an episode

In the previous section we used an episode length of 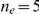 to construct the episode networks. That means we defined an episode as 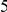 consecutive cycles as the nodes in our networks. In this section we use the quantitative procedure introduced in the methods section that allows to determine the *effective length* of an episode automatically. Our results will show for different systems that in general 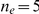 is a good choice for the effective length of an episode.

In order to determine the effective length of an episode we start with a time series of a fixed length 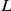 and construct for various values of 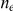 the corresponding episode networks. According to the definition of *effective length*, we need to identify the value 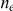 from which on the characteristics of the networks change. Per definition, this is the point of the first decrease of the maximal global clustering coefficient.

**Figure 7:**
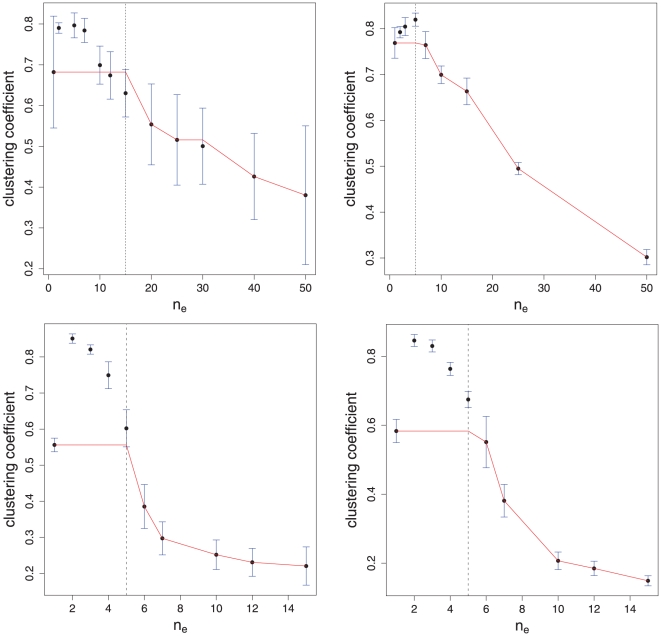
Efficient length of an episode. First row: Rössler system. Left: 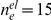. Right: 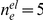. Second row: Lorenz equations. Left: 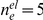. Right: 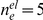.

The first time series we study is again from a Rössler systems with the same parameters as in Eqn. 4–6. The left Fig. 7 at the top shows our results averaged over 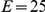 independent time series. Here the black dots correspond to the mean value of the clustering coefficient and the error bars give its standard deviation. Interestingly, the mean clustering coefficient does not only increase in the first step from 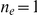 to 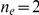 but its standard deviation decreases considerably. This indicates a stabilizing effect of longer episodes on the constructed episode networks. That means despite the fact that 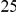 different time series have been used, the resulting networks become more similar to each other for 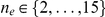 than for other values of 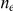. This kind of robustness is desirable because the networks should reflect characteristics of the underlying dynamical system, rather than only of the individual time series used, which provides merely a sample thereof. In order to provide a quantitative cut-off value of the effective episode length, we use algorithm 1 to calculate the function 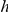. The result of this is included in Fig. 7 showing the function 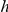 in red. The maximal value of 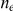 for which 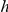 is maximal is indicated as a vertical line, corresponding to an effective length of 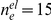.

For the above analysis we used time series of a fixed length 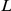, and constructed different episode networks for different values of 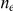. Due to the fact that the time series is of fixed length 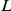, the networks constructed for larger values of 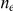 are smaller (consist of fewer nodes) than the networks for smaller 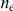 values. In order to study if this effects the obtained results we repeated the above analysis, however, this time we keep the size (number of nodes) of each constructed episode network fixed. That means we need to generate larger time series for larger values of 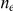. More precisely, in order to maintain a constant size of the episode networks, the length of the time series used to construct an episode network for 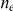 needs to be of length 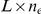. The right Fig. 7 at the top shows the results of this analysis, again averaged over 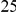 independent simulations. Due to the larger length of the time series used for this new analysis the variances are in general smaller. This leads to a more conservative estimate of 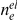 which is in this case 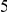.

As a second example we determine the effective episode length for time series from Lorenz equations, see Eqn. 9 to 11. As time series we use again the x-component of the Lorenz equations. The results of our analysis are shown in the second row in Fig. 7. The obtained results are similar to the Rössler system with the difference that for the Lorenz equations the determined effective length is 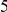 for the fixed length time series (left figure) and the fixed size episode networks (right figure). As a general observation from our studies we note that there is always an increase in the clustering coefficient at the first step regardless of the considered time series. That means it is always beneficial to use an episode length 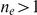 to construct episode networks.

The above analysis shows that the efficient episode length 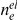 resulting from a ‘fixed length time series’ can be the same as from a ‘fixed size episode networks’, but it does not have to. Due to the fact that the variability the in latter analysis is usually smaller the resulting estimates for 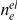 are more conservative and, hence, preferred. From this follows that it is advisable to use no episode lengths longer than 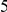 because this will lead to changing structural characteristics of the constructed networks. Using smaller values than 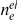 is in principle possible, however, the resulting networks are larger. That means these networks contain a similar amount of information as the episode network constructed for 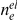, but they are larger because they consist of more nodes due to smaller episode lengths. Usually, larger networks consume more time for their analysis. For this reason, it is desirable to have the smallest networks possible that contain the same information.

### Application: EEG data

Finally, we demonstrate that episode networks are useful in the practical analysis of time series data. We use electroencephalographic (EEG) time series data which measure the electrical activity of the brain [51], [52]. From the total dataset available for a study that contains recordings from a 128-channel amplifier system [53], we select three different types of data which come from extracranial and intracranial recordings. The first type corresponds to surface EEG recordings of control patients. The second and third type are from intracranial recordings from presurgical patients measured in the hippocampus formation. Type 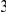 represent only seizure free intervals, whereas type 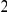 measures seizure activity.

For these three data types, we use a total of 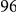 single-channel recordings of 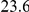 seconds duration. From these data we create 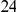 time series of length 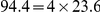 seconds to obtain time series of a sufficient length. That means in total we construct for each of the three different data types 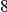 individual episode networks and average over the obtained results. This mimics three (small) populations of patients in order to estimate the variability within these populations. For constructing the episode networks we use as efficient length 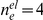. The results from this analysis for the average path length and the clustering coefficient are shown in table 1. The variables 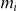 refer to the mean values whereas 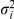 provide the corresponding variance.

**Table 1:** Numerical results obtained for the EEG dataset [53].

measure	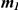	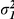	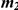	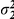	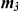	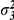
average path length	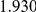	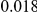	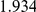	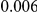	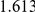	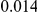
clustering coefficient	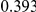	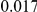	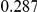	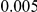	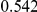	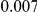

The index of the measures refers to one of the three data types used. 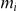 corresponds to the mean value of the measure and 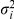 to its variance. The indices correspond to: 1: control group; 2: patients - seizure free intervals; 3: patients - seizure intervals.

It is interesting to see that the average path length is quite similar for the first two data types. Only data type three can be clearly distinguished. The situation is different for the clustering coefficient. Here there is a clear separation between all three data types. We confirm this impression statistically by a two-sample t-test [54] comparing the mean values of the clustering coefficients. For all three tested cases, we obtain a statistically significant result assuming a significance level of 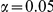. That means, the null hypothesis assuming equal means among the groups is rejected. The selected significance level of 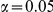, which implies an expected false positive rate of 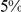, appears reasonable in the given context. It is interesting to note that the variance of all variables is quite small indicating that each of the 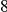 networks comprising a population is representative for the other networks of the population because the measured properties are very similar. This is a desirable property, because it allows an experimental design with small sample sizes involving only a few patients. Especially, for invasive procedures this is an advantage because such procedures are usually accompanied by severe discomfort for the patients and costs for Public Health.

**Figure 8:**
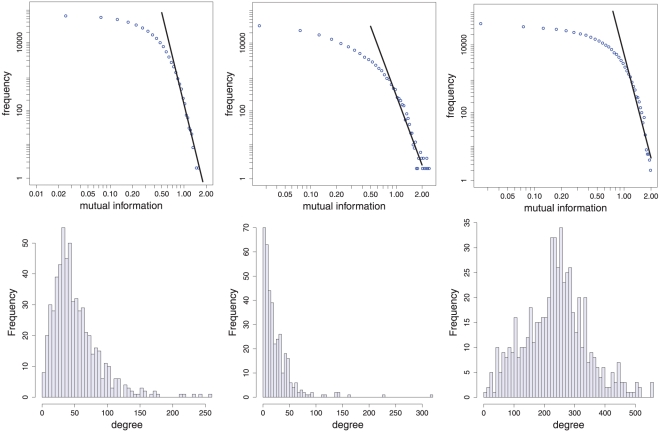
Results for the EEG dataset [**53**]. Left column: data type 1 (control group). Middle column: data type 2 (patients - seizure free intervals). Right column: data type 3 (patients - seizure intervals). The first row shows the distribution of mutual information values. The second row shows the histogram of degrees.

In the top row in Fig. 8 we show the distribution of mutual information values for three episode networks, one for each of the three data types. From these figures one gets the impression that all networks follow a power law distribution in the tails for their corresponding mutual information values. This is quantitatively confirmed by the statistical test suggested in [55] resulting in the p-values 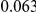, 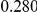 and 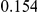 for the cut-off values 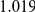, 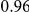 and 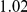. That means the tails of these mutual information distributions follow a power law, 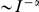, and are not only long tails as for the Rössler system and the Duffing map.

In the second row in Fig. 8 we show the histograms of the degrees for these networks. It is interesting to see that despite the similarity of the distribution of mutual information values for all networks, their degree distributions are remarkably dissimilar. The network from the control patients (left most figure) seems to be between the other two networks with respect to the degree distribution. Quantitatively, this is confirmed by the mean value of the distributions which are 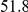 (left figure - data type 1), 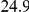 (middle figure - data type 2) and 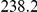 (right figure - data type 3). This observation is plausible because it means that during a seizure the EEG time series becomes more irregular and, hence, the similarity between different episodes is reduced. This leads to a reduction in the connectivity in the episode networks, which can be directly observed in the degree distribution in the middle Fig. 8. In contrast, during seizure free intervals the episodes become more similar leading to an increase in the connectivity of the episode network. However, it is less obvious that episodes of patients for time series of seizure free intervals (data type 3) are more similar than for control patients (data type 1). One implication from this observation is that even for seizure free intervals the EEG activity of such patients is considerably different compared to control patients. This might be a property useful for diagnostic purposes.

## Discussion

In summary, in this paper we introduced a method to construct networks from pseudoperiodic time series, which we called *episode networks*. Our method is parametric allowing for the adjustment of the length of an episode, which defines the nodes in the network, and, hence, allows for the modification of the size of a network. We demonstrated, numerically, that it is always beneficial to use an episode length longer than one cycle and we defined the *effective* length of an episode as the solution of an optimization problem for the measure 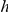. The measure 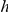 was defined reflecting the average global clustering coefficients of episode networks and their variability with respect to a population of episode networks. Using the *optimal* value 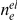 as an episode length to construct an episode network leads to the smallest network that contains, approximately, the same information as larger episode networks, because networks constructed for an episode length smaller than 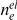 lead to similar values of 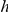, but larger networks. Another novel feature of our construction method is that it employes estimates of mutual information values to assess the similarity between different intervals of the time series, to construct the connectivity among the nodes. This allows to capture nonlinearities that are doubtlessly present in pseudoperiodic time series. From the application of our network construction method to data from EEG experiments we found that the episode networks corresponding to different groups (patients or control) capture discriminative information from the underlying time series allowing a clear distinction from each other. Furthermore, the revealed differences in the degree distributions might be useful for diagnostic purposes. However, additional studies are necessary with data from independently conducted EEG experiments to establish the robustness of the obtained results with respect to varying experimental settings and protocols. This could also provide valuable insights into the experimental design of EEG experiments and differences among them, leading to a stratification in the way that our method could be applied to data from a certain subgroup of experimental designs.

From the above discussion one might feel tempted to ask if the traditional methodology for time series analysis [56], [57] should be substituted by the structural analysis of networks constructed from an underlying time series. However, we do not think that it is necessary to substitute one approach by the other, instead, the analysis of networks constructed from time series data should be considered as a valuable addition to the standard methodology for time series analysis. Interestingly, from a statistical perspective there is, in fact, nothing special about the usage of networks as representation of the data. In the context of gene expression data this has been demonstrated by [58], [59] showing that a correlation matrix can be interpreted as a weighted network that contains meaningful information about the interactions among genes. If seen from this angle, networks form an integral part of many methods in multivariate analysis [60].

There are several other methods that have been introduced in recent years to construct a network from time series data [30], [31], [33] following the spirit of [34]. Due to the fact that time series data are available in many different fields, e.g., biology, chemistry, physics, medicine or the social sciences, methods to convert these data into networks in order to enable a subsequent analysis are certainly of interest for a large variety of different application domains. Specifically, in molecular biology, the concentration of mRNAs is measured by DNA microarrays allowing genome-wide expression levels of all genes to be obtained. For this reason, periodic processes like the cell cycle or the circadian rhythm could be studied by means of *episode networks* [61]–[63]. From a theoretical perspective, it would be interesting to investigate in-depth the structural properties of *visibility*, *cycle* and *episode networks* to gain a thorough understanding of the coupling between their features and the properties of the time series, with respect to their generation processes.
